# Mediating roles of patient safety knowledge and motivation in the relationship between safety climate and nurses’ patient safety behaviors: a structural equation modeling analysis

**DOI:** 10.1186/s12912-022-01123-6

**Published:** 2022-12-01

**Authors:** Ja-Kyung Seo, Seung Eun Lee

**Affiliations:** 1 Department of Psychology, Graduate School, Yonsei University, Seoul, South Korea; 2 College of Nursing, Mo-Im KIM Nursing Research Institute, Yonsei University, 50-1 Yonsei-ro, Seodaemun-gu, Seoul, 03722 South Korea

**Keywords:** Patient safety, Behavior, Korea, Nursing, Safety management

## Abstract

**Background:**

Few studies have examined the relationship between patient safety climate and two forms of patient safety behavior (i.e., safety compliance and safety participation) among nurses. Better understanding of factors contributing to nurses’ safety behaviors could enhance patient safety. Therefore, this study aimed to examine the effect of patient safety climate on nurses’ patient safety behavior and to explore whether patient safety knowledge and motivation mediate this relationship.

**Methods:**

This correlational, cross-sectional study used survey data from 1,053 staff nurses working at a general hospital located in a metropolitan area of South Korea. Structural equation modeling was employed to test a hypothesized multiple mediation model that was guided by Griffin and Neal’s model of safety performance.

**Results:**

The results indicated that patient safety climate was directly related to both patient safety compliance behavior (β = 0.27, *p* < 0.001) and patient safety participation behavior (β = 0.25, *p* < 0.001). Concerning indirect effects, patient safety climate was associated with patient safety compliance behavior through both patient safety knowledge (β = 0.26, *p* < 0.001) and patient safety motivation (β = 0.04, *p* = 0.038), whereas patient safety climate was related to patient safety participation behavior only through patient safety knowledge (β = 0.27, *p* < 0.001) and not through patient safety motivation (β = 0.00, *p* = 0.985).

**Conclusion:**

Based on this study’s findings, building an organizational climate focused on patient safety is vital for improving nurses’ patient safety behavior. Improving an organization’s patient safety climate could promote both safety knowledge and motivation in nurses and thereby potentially enhance their patient safety behavior. Hence, healthcare organizations should implement practical interventions to improve their patient safety climate. Also, nursing management interventions designed to transfer patient safety knowledge to nurses would be particularly effective in improving their safety behavior.

## Background

Patient safety is a fundamental component of and priority for global healthcare systems [1], and safety behavior represents a major international concern in healthcare organizations because it is critical for enhancing patient safety [2]. In healthcare, safety behavior consists of actions and performance that can prevent adverse events during care provision [3] and includes two behavioral components—safety compliance and safety participation [3–6]. Safety compliance refers to mandatory safety activities that individuals must conduct to maintain workplace safety, including adherence to standard procedures and guidelines [4, 7]. For example, wearing protective equipment is an essential safety compliance activity for infection control and prevention. Safety participation describes behaviors related to participating in voluntary safety activities and making extra efforts to improve safety [3, 5, 8]. While safety participation behaviors may not directly contribute to safety in work areas, they contribute to creating an environment that supports safety [7] and reduces adverse incidents [2].

As nurses comprise the largest healthcare workforce and have the closest proximity to patients, their patient safety compliance and participation behaviors may directly affect patient safety in healthcare organizations. Despite the importance of nurses’ role in patient safety, few studies have examined the precursors of nurses’ patient safety behaviors [9, 10]. In contrast, the organizational literature [5, 6] identifies safety climate as one factor influencing employees’ safety behaviors; recent organizational research has reported significant associations between safety climate and safety behaviors among non-healthcare workers such as Korean hotel employees and Vietnamese manufacturing workers [11, 12]. Similarly, previous healthcare research showed a significant association between safety climate and the safety behaviors of Dutch medical residents [3]. Although nursing research in this area is sparse, we posit that a comparable relationship may exist among nurses.

Safety climate refers to the perceived value that an organization places on safety [5]. When nurses perceive that their organization supports patient safety by demonstrating open communication, supportive leadership, and adequate resources, they in turn are more likely to engage in patient safety behaviors [6], as they perceive that the climate is conducive for such behaviors [13]. Additionally, when organizations actively show their support for safety, nurses may be willing to reciprocate by voluntarily engaging in safety activities beyond their formal job descriptions [12, 14, 15]. In this study, our focus is safety climate and safety behaviors pertaining to patient safety. Thus, we hypothesized that nurse-perceived patient safety climate is positively associated with nurses’ patient safety behaviors as follows:

- Hypothesis 1a. Patient safety climate has a positive relationship with nurses’ patient safety compliance behavior.
- Hypothesis 1b. Patient safety climate has a positive relationship with nurses’ patient safety participation behavior.

Furthermore, Griffin and Neal’s model of safety performance suggests both distal and proximal determinants of safety behaviors [4]. This link between safety antecedents and safety behaviors is grounded in Campbell et al.’s theory of job performance, which suggests that distal antecedents of performance (e.g., organizational climate) influence job performance by increasing proximal determinants such as knowledge and motivation to perform [16]. Safety climate, a subset of organizational climate, can be viewed as a distal factor influencing safety behaviors; thus, this relationship may be mediated by safety knowledge and motivation [4–6].

Regarding safety knowledge, an individual must understand how to perform work safely to be able to comply with existing safety procedures; as behavioral decisions often result from a reasoned process, a link between knowledge and behavior is likely [5, 8]. Moreover, safety knowledge has been found to significantly predict both safety compliance and safety participation [4, 17]. For instance, level of knowledge about occupational and health safety was significantly correlated with the safety behaviors of Chinese industrial workers [8]. Thus, in the nursing context, we posit that patient safety knowledge may mediate the association between patient safety climate and the two forms of patient safety behavior—safety compliance and participation—among nurses.

Safety motivation refers to an individual’s willingness to engage in safety behaviors and the importance that individuals place upon the expected safety outcome [7]. That is, in directing, stimulating, and sustaining action, safety motivation psychologically prompts employees to comply with safety regulations and voluntarily participate in safety activities [18]. Empirical evidence has shown that safety motivation is critical for establishing workplace safety behaviors in various industrial and organizational contexts [11, 19, 20]. Although researchers have examined the mediating role of safety motivation in the relationship between safety climate and safety behaviors, their results have been mixed. Some studies have reported a significant association between safety motivation and both forms of patient safety behavior [4, 11, 21]; however, another study found no significant lagged effect of safety motivation on safety compliance behavior among healthcare employees [7]. These inconsistent results warrant further empirical investigation. Hence, we examined a mediation model in which patient safety motivation is a proximal precursor of patient safety behavior that mediates the association between patient safety climate and safety behaviors. Accordingly, our second and third sets of hypotheses were as follows:

- Hypothesis 2a. Patient safety knowledge mediates the positive relationship between patient safety climate and nurses’ patient safety compliance behavior.
- Hypothesis 2b. Patient safety knowledge mediates the positive relationship between patient safety climate and nurses’ patient safety participation behavior.
- Hypothesis 3a. Patient safety motivation mediates the positive relationship between patient safety climate and nurses’ patient safety compliance behavior.
- Hypothesis 3b. Patient safety motivation mediates the positive relationship between patient safety climate and nurses’ patient safety participation behavior.

In sum, this study aimed to identify the underlying mechanisms linking patient safety climate to patient safety behaviors of Korean nurses using a research model that focused on two types of safety behaviors—safety compliance and safety participation [4, 7].

## Methods

### Design and sample

This cross-sectional, correlational study used de-identified secondary data from 1,053 staff nurses working in a non-profit, acute care, teaching hospital in a major metropolitan area of South Korea. In October and November 2021, following the Agency for Healthcare Research and Quality guidance [22], the original paper–pencil surveys for the biannual organizational safety culture assessment were distributed to all staff through designated points of contact within the hospital. All staff members were informed that survey participation was voluntary and their responses will be kept confidential. Accordingly, 1,084 nurses completed the survey; of these, 1,053 were staff nurses who met the inclusion criteria of this study. We excluded nurse managers, directors, and administrators due to the potentially differing perceptions of patient safety climate arising from their positions [23].

According to Fritz and MacKinnon [24], the sample size of 1,053 was adequate for mediation analysis using a bias-corrected bootstrap method to achieve a power of 0.8 for identifying the small effect of a predictor on a mediator as well as the small effect of the mediator on an outcome variable after accounting for the mediator.

### Measures

The survey included scales that measured the key study variables. The total mean score for each scale was computed, with a higher score indicating a higher level of the construct. Also, the survey collected respondents’ demographic information: gender, age, employment status, work unit, hospital tenure, and unit tenure.

#### Outcome variables

Items from the Safety Performance Scale developed by Neal et al. [5] were revised to assess patient safety compliance behavior and patient safety participation behavior. Both scales include three items assessed on a 5-point Likert scale ranging from 1 (strongly disagree) to 5 (strongly agree). An example item for compliance behavior is, “I use the correct patient safety-related procedures for carrying out my job,” and a sample item for participation behavior is, “I voluntarily carry out tasks or activities that help improve patient safety.” For the study sample, exploratory factor analysis (EFA) with principal axis factoring yielded a one-factor model for each scale. The one-factor structure explained 78.6% of the total variance for compliance behavior and 79.13% for participation behavior, with factor loadings ranging from 0.76 to 0.88 for the two scales. In this study, the response to patient safety compliance behavior and patient safety participation behavior had Cronbach’s alphas of 0.86 and 0.87, respectively.

#### Predictor variables

Patient safety climate was assessed using the 31-item Korean version of the Hospital Survey on Patient Safety Culture 2.0 (K-HSOPSC 2.0), which has demonstrated good reliability and validity [25]. An example item is “This unit regularly reviews work processes to determine if changes are needed to improve patient safety.” Responses were measured on a 5-point scale ranging from 1 (strongly disagree or never) to 5 (strongly agree or always) depending on the item, and the total mean score was calculated. Cronbach’s alpha for the scale was 0.91 for the study sample.

#### Mediating variables

Patient safety knowledge and patient safety motivation were assessed using revised items from Neal et al. [5]. Patient safety knowledge was measured using three items that assessed nurses’ knowledge about patient safety practices and procedures, and patient safety motivation was measured using three items that assessed the extent to which nurses viewed patient safety as an important part of their work. For both scales, nurses responded on a 5-point scale ranging from 1 (strongly disagree) to 5 (strongly agree). An example item for patient safety knowledge is, “I know how to maintain or improve patient safety,” while a sample item for patient safety motivation is, “I feel that it is worthwhile to put in effort to maintain or improve patient safety.” For the study sample, EFA with principal axis factoring yielded a one-factor model for each scale. The one-factor structure explained 82.9% of the total variance for patient safety knowledge and 84.3% for patient safety motivation, with factor loadings ranging from 0.76 to 0.93 and from 0.75 to 0.95 for safety knowledge and safety motivation, respectively. In this study, the response to patient safety knowledge and patient safety motivation had Cronbach’s alphas of 0.90 and 0.91, respectively.

### Data analysis

Prior to model testing, SPSS version 25.0 was used to calculate descriptive statistics, Pearson’s bivariate correlations, and reliability estimates. The measurement model and hypothesized multiple mediation model were tested using structural equation modeling (SEM) performed with Mplus version 8.6. The models were estimated with the robust maximum likelihood method, and the results were examined using indices and recommended cutoff values for model fit, including the standardized root mean square residual (SRMR < 0.08), comparative fit index (CFI > 0.90), Tucker-Lewis Index (TLI > 0.90), and root mean square error of approximation (RMSEA < 0.08) (Wang et al., 2011). Work unit and hospital tenure were controlled for in the SEM analysis because previous studies had reported that nurses in different work units and with differing hospital tenures showed differences in relationships between patient safety climate perceptions and patient safety compliance and participation behaviors [26, 27]. Finally, we implemented bootstrapping with 10,000 bootstrap samples and 95% bias-corrected confidence intervals (CI) to test the significance of direct and indirect effects [28].

## Results

### Descriptive statistics

Most study participants were women (*n* = 990, 94.6%), and more than half (*n* = 606, 57.9%) were in the 20–29 year age group. The mean length of tenure in the current unit was 3.9 years (*SD* = 4.4), and the mean length of hospital tenure was 7.1 years (*SD* = 7.4). The great majority of participants (*n* = 964, 95.3%) had a permanent, full-time position. Participants’ work units included medical, surgical, and medical-surgical (*n* = 454, 44.0%); critical care (*n* = 188, 18.2%); perioperative (*n* = 65, 6.3%); and other (*n* = 346, 33.5%) units. The other units included emergency department, rehabilitation, pediatric, obstetric, psychiatric, outpatient, urology, and labor and delivery units as well as multiple units.

Descriptive statistics for key study variables and their correlations are shown in Table 1. Bivariate correlation results showed that nurses’ perception of patient safety climate was positively associated with both patient safety knowledge (*r* = 0.50, *p* < 0.001) and motivation (*r* = 0.44, *p* < 0.001). Patient safety climate was also positively related to patient safety compliance behavior (*r* = 0.55, *p* < 0.001) and participation behavior (*r* = 0.51, *p* < 0.001). Moreover, patient safety knowledge was positively and strongly associated with both patient safety compliance behavior (*r* = 0.70, *p* < 0.001) and participation behavior (*r* = 0.64, *p* < 0.001). Finally, patient safety motivation was also positively related to both patient safety compliance behavior (*r* = 0.55, *p* < 0.001) and participation behavior (*r* = 0.43, *p* < 0.001).

**Table 1:** Correlations, descriptive statistics, and Cronbach’s alpha for study variables

Variable	1	2	3	4	5
1.Patient safety climate	—				
2.Patient safety knowledge	0.50**	—			
3.Patient safety motivation	0.44**	0.54**	—		
4.Patient safety compliance behavior	0.55**	0.70**	0.55**	—	
5.Patient safety participation behavior	0.51**	0.64**	0.43**	0.72**	—
*M*	3.53	3.84	4.22	3.89	3.74
*SD*	0.42	0.57	0.59	0.57	0.63

*M* mean, *SD* standard deviation. ^*^p < .05, ^**^p < .01

### Hypothesis testing

Before hypothesis testing, variance inflation factors (VIF) between the predictor and mediator variables were examined. VIFs ranged from 1.40 to 1.61, indicating no potential multicollinearity [29]. In terms of normality, the skewness or kurtosis levels of the five study variables ranged from -0.42 to 0.36, well below the threshold of an absolute value of 2 [30]. Next, the measurement model was examined using confirmatory factor analysis (CFA) to evaluate how well the observed items represented the latent variables and how distinct the key constructs were from one another. The measurement model showed good data fit, with fit indices of χ^2^(199) = 1,129.26, CFI = 0.93, TLI = 0.92, RMSEA = 0.07, and SRMR = 0.05. All items’ factor loadings to their respective latent factors were statistically significant (*p* < 0.001).

**Fig. 1:**
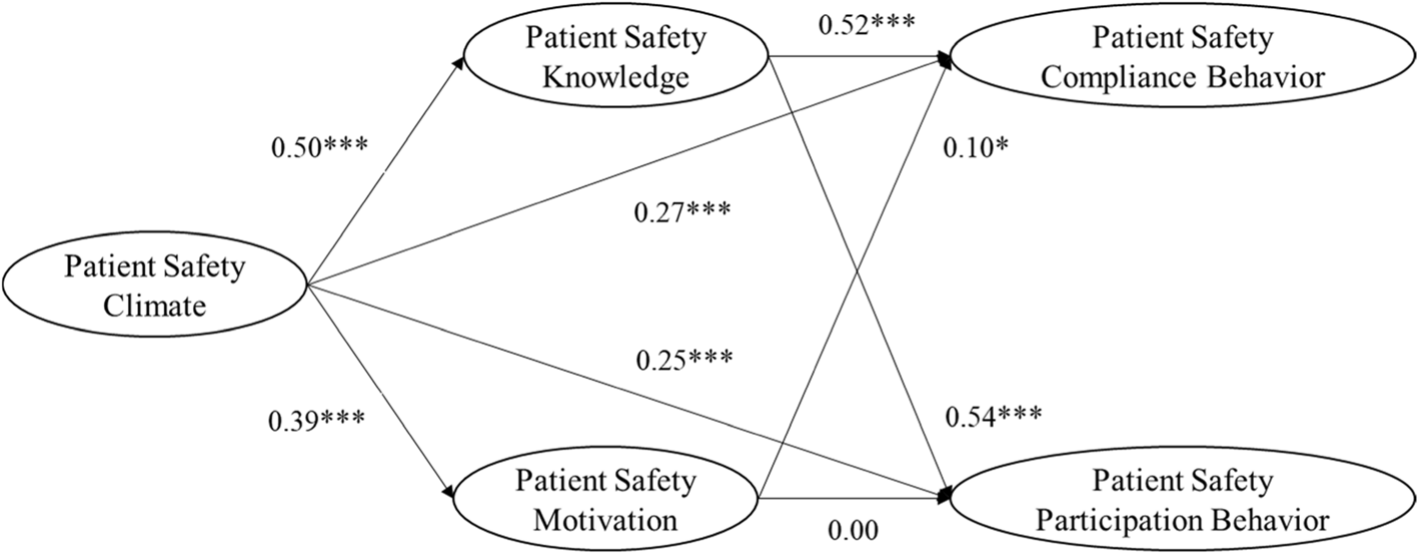
Results of multiple mediation analysis. Standardized coefficients of direct and indirect effects of patient safety climate on safety compliance behavior and safety participation behavior through safety knowledge and safety motivation. Hospital tenure and unit dummy variables were controlled for in the model. *p < 0.05, ^**^ *p* < 0.01, ^***^ *p* < 0.001

Our hypothesized model (see Fig. 1) also showed good fit to the data, with fit indices of χ^2^(399) = 1,720.22, CFI = 0.91, TLI = 0.90, RMSEA = 0.06, and SRMR = 0.05. The results of SEM analysis revealed that patient safety climate had positive and significant direct effects on patient safety compliance (β = 0.27, 95% CI [0.17–0.37]) and participation behavior (β = 0.25, 95% CI [0.15–0.34]) (see Table 2). We also found that patient safety compliance behavior was significantly associated with both patient safety knowledge (β = 0.52, *p* < 0.001) and motivation (β = 0.10, *p* = 0.023), but the effect of patient safety knowledge was stronger. Of the mediator variables, only patient safety knowledge was significantly related to patient safety participation behavior (β = 0.54, *p* < 0.001); patient safety motivation showed no significant relationship to participation behavior.

**Table 2:** Standardized direct and indirect effects of hypothetical model

Path	β	*p*	95% CI [Lower, Upper]
Direct effect			
Patient safety climate -> Patient safety compliance behavior	0.273	< 0.001	[0.167, 0.372]
Patient safety climate -> Patient safety participation behavior	0.251	< 0.001	[0.153, 0.344]
Indirect effect			
Patient safety climate -> Patient safety knowledge -> Patient safety compliance behavior	0.258	< 0.001	[0.195, 0.325]
Patient safety climate -> Patient safety motivation -> Patient safety compliance behavior	0.039	0.038	[0.006, 0.080]
Patient safety climate -> Patient safety knowledge -> Patient safety participation behavior	0.268	< 0.001	[0.201, 0.340]
Patient safety climate -> Patient safety motivation -> Patient safety participation behavior	0.00	0.985	[-0.033, 0.037]

*CI* bias-corrected confidence interval

The 95% CIs for the parameter estimates were calculated using 10,000 data samples from the raw data. The indirect effects of patient safety climate on patient safety compliance behavior through patient safety knowledge (β = 0.26, 95% CI [0.20–0.33]) and motivation (β = 0.04, 95% CI [0.01–0.08]) were significant. However, the indirect effect of patient safety climate on patient safety participation behavior was significant only when mediated by patient safety knowledge. Specifically, patient safety climate was significantly associated with patient safety participation behavior through patient safety knowledge (β = 0.27, 95% CI [0.20–0.34]) but not through patient safety motivation (β = 0.00, 95% CI [-0.03–0.04]).

## Discussion

This study explored the relationship between patient safety climate and patient safety behavior and investigated the mediating roles of patient safety knowledge and motivation in a sample of Korean nurses. Although determinants of employee safety behavior have been identified in various industries, such investigations with nurses have been sparse. Safety research needs to address industry-level differences, as each industry has its own distinct job descriptions, work atmospheres, and safety management systems [10, 18]. Hence, our study explored whether an increased patient safety climate was related to improved patient safety behaviors among nurses in a healthcare context. Greater understanding of this mechanism can provide insights to support development of measures for improving patient safety.

As shown in Table 2 and Fig. 1, the significant direct effects indicate that patient safety climate directly increases both patient safety compliance and participation among nurses. Thus, if nurses perceive that their organization has a positive orientation toward safety in their daily interactions [18, 31], they may be more willing to display safety behavior in order to meet their organization’s safety expectations [9]. Furthermore, our results suggest that patient safety climate has an independent effect on nurses’ patient safety behavior even without producing changes in safety knowledge or motivation in the process.

Study results partially support our hypotheses related to the two mediator variables. As we hypothesized, patient safety knowledge was a significant mediator in the relationship between patient safety climate and patient safety behavior. Although safety climate was positively related to both safety knowledge and safety motivation, the bootstrapping analyses indicated that safety knowledge alone mediated the relationship between safety climate and safety behaviors. This result supports previous findings of a positive association between safety knowledge and safety behavior [5, 8, 20] but also highlights the particular importance of safety knowledge in increasing safety behavior among Korean nurses. Safety knowledge may serve as a job resource supporting nurses’ engagement in safety behavior [32]. Our findings indicate that interventions targeting patient safety knowledge—including regular training and provision of clear guidelines regarding patient safety—may be more effective than patient safety motivation strategies (such as incentive schemes) in improving nurses’ patient safety behavior.

Contrary to our expectations, the indirect path from patient safety climate to patient safety participation behavior through patient safety motivation was not significant. Also, although the mediating effect of safety motivation between safety climate and patient safety compliance behavior was significant, the effect was quite small compared to the mediating effect of patient safety knowledge. This result is inconsistent with previous organizational research findings that safety motivation was significantly related to both forms of employee safety behavior in non-healthcare industries such as the hotel, chemical, and mining industries [4, 11, 20] and with claims emphasizing the role of motivation in initiating safety performance behavior [5, 7, 18]. After considering the potential reasons for this discrepancy, we suspect that the nonsignificant effect of patient safety motivation on patient safety behaviors in our sample may have been due to our use of a motivation scale containing only three items. In our study, participants’ mean patient safety motivation score was 4.22 on a 5-point scale, whereas their mean patient safety knowledge score was 3.84; thus, participants tended to report their safety motivation to be fairly high, limiting the variance for this variable. Future researchers should develop and employ measures that address a wide range of safety motivation behaviors [7], and more research is needed to investigate the effects of safety motivation on safety behavior among nurses and other healthcare professionals.

To the best of our knowledge, this study is the first to examine the direct and indirect effects of patient safety climate on nurses’ patient safety behavior and to explore the mediating roles of patient safety knowledge and motivation in a Korean healthcare context. Overall, our results illustrate the importance of organizational context to nurses’ patient safety behavior and to individual improvements in their safety knowledge and motivation [13]. Thus, our study addresses an important gap in the nursing and patient safety literature by identifying a potential mechanism for nurses’ patient safety behavior. Lastly, our results not only generally support existing theoretical assumptions regarding the relationship between safety climate and safety behavior but also include unexpected findings regarding safety motivation. Researchers should further examine the role of safety motivation in pursuing ways to improve nurses’ patient safety behavior.

This study has several limitations that should be noted. First, our analysis of data from a single hospital in South Korea may limit the generalizability of our study findings to other populations. Thus, further research should be conducted with other nurse populations in various settings. Second, this study had a cross-sectional design, and so no conclusions can be drawn regarding causal relationships between variables. Longitudinal studies are needed to determine causal associations between study variables and to further investigate how organizational factors contribute to nurses’ patient safety behaviors in the long term. In particular, researchers should examine the possibility that the relationship between patient safety motivation and patient safety behavior could be reciprocal over time, as suggested by Neal & Griffin [7]. Third, the study data were collected entirely with self-reported measures. Although EFA results for four scales (i.e., those for patient safety knowledge, motivation, compliance behavior, and participation behavior) supported each factor structure, and although CFA results indicated good data fit for the measurement model, reporting bias may be present. Along with self-reported measures, future research may use observational methods to objectively assess nurses’ patient safety behaviors in order to minimize the potential for bias.

## Conclusion

Determining how to promote nurses’ safety compliance and participation behaviors is critical, especially during the COVID-19 pandemic, when adherence to stringent safety rules and procedures is required [8]. Additionally, understanding whether and how patient safety climate is associated with nurses’ patient safety behaviors would allow us to identify practical strategies in order to improve patient safety outcomes [33]. Our findings for a sample of Korean nurses indicate that improving the patient safety climate within healthcare organizations is a viable strategy for enhancing nurses’ patient safety behavior. The study results also explain how patient safety climate is related to patient safety compliance and participation behavior through the mediating roles of patient safety knowledge and motivation. Therefore, interventions aimed at fostering an organizational safety climate could have a positive impact on patient safety compliance and participation behavior among nurses.

## Data Availability

The datasets generated and/or analysed during the current study are not publicly available due to them containing information that could compromise research participant consent but are available from the corresponding author on reasonable request.
